# The efficacy of cognitive remediation in children and adolescents: results from a new meta-analysis.

**DOI:** 10.1192/j.eurpsy.2023.65

**Published:** 2023-07-19

**Authors:** A. Moscoso

**Affiliations:** Child and Adolescent Psychiatry, Hopital Universitaire Robert Debré, Paris, France

## Abstract

**Abstract:**

This presentation will share data from a new meta-analysis of RCT studies using cognitive remediation to treat children and adolescents with ADHD. For this matter, 25 studies were accessed, and results will focus on outcomes immediately after the end of the program and in follow-up. Outcomes include subjective measures of functioning (through validated scales), neuropsychological measurements and academic performance. Comparisons regarding length of training, intensity, concomitant use of pharmacological treatments will also be provided. These findings will build on previous meta-analysis on the issue that were performed in the past.

**Disclosure of Interest:**

None Declared

